# What makes people grow? Love and hope

**DOI:** 10.1186/s40101-023-00330-7

**Published:** 2023-07-13

**Authors:** Barry Bogin

**Affiliations:** 1 UCSD/Salk Center for Academic Research and Training in Anthropogeny (CARTA), San Diego, USA; 2 School of Sport, Exercise & Health Sciences, Loughborough University, Loughborough, UK; 3 University of Michigan-Dearborn, Dearborn, USA; 4 Diversity Scholars Network, Ann Arbor, USA

**Keywords:** Biocultural reproduction, SEPE, Secular trend, Community effects, Strategic growth

## Abstract

**Background:**

Hope and love are popular themes of literature and art in many human societies. The human physiology of love and hope is less well understood. This review presents evidence that the lack of love and/or hope delays growth disturbs development and maturation and even kills.

**Main body:**

Love and hope intersect in promoting healthy human development. Love provides a sense of security and attachment, which are necessary for healthy physical, cognitive, and emotional development. Hope provides a sense of optimism and resilience in the face of adversity. Loving relationships can foster a sense of hope in individuals and in society by providing support systems during difficult times. Similarly, having a sense of hope can make it easier to form loving relationships by providing individuals with the confidence to connect with others. Hope and love are the fundamental basis of human biocultural reproduction, which is the human style of cooperation in the production, feeding, and care of offspring. Examples are given of the association between human growth in height with love and hope, including (1) the global “Long Depression” of 1873–1896, (2) “hospitalism” and the abuse/neglect of infants and children, (3) adoption, (4) international migration, (5) colonial conquest, and (6) social, economic, and political change in Japan between 1970 and 1990.

**Conclusion:**

Overall, this review suggests that love and hope are both critical factors in promoting healthy human development and that they intersect in complex ways to support emotional well-being.

## Background

*Hope—Hope in the face of difficulty. Hope in the face of uncertainty. The audacity of hope!* ~ Barack Obama, 2004 keynote address to the Democratic Convention.

*The human mind can cope with anything but the loss of hope.* ~ Elizabeth Luard, from her book *Family Life*, 1996, p. 28).

*I think that HOPE is the single most important emotional factor for all aspects of human growth, development, and maturation*. B Bogin, from the lecture ‘Hope, despair, and hope again: the intersecting cycles for healthy human growth and development’ delivered at Durham University 23 February 2023.

As a child in the 1950s, my mother said to me: “There are starving children in Europe. Eat your supper, otherwise you will not grow.” This seemed right to me at the time. During and after World War II, Europe did experience starvation in the Netherlands, in Greece, in eastern Europe, and the Soviet Union and defeated Germany. Food rationing was imposed everywhere. In the USA, it started in 1942 and most food rationing ended in December 1945, but sugar rationing continued until June 1947. My mother was a teenager during those years and experienced food rationing directly. In victorious Britain, food rationing did not end until midnight on 4 July 1954, when restrictions on the sale and purchase of meat and bacon were lifted. That was 9 years after the end of the war. Photographs I saw in *Life magazine* and elsewhere (https://www.magnumphotos.com/newsroom/society/david-seymour-children-of-europe/) in the late 1950s and early 1960s depicted war refugees, holocaust survivors, and US conscientious objectors to military service who “volunteered” for The Minnesota Starvation Experiment [1], www.bbc.co.uk/news/magazine-25782294). To my child mind, these images evoked hunger as the people were thin and seemed small. My mother had to be right—if those people had more food to eat, then they would be bigger.

Young people do need food to grow. They also require adequate housing and sanitation, health care, education, and many other decent standards of material and social living conditions. These needs have been widely promulgated in writing since Sumerian Mesopotamia, about 3500 BP [2]. In contrast, the essential emotional factors required for healthy growth and development have often been downplayed or ignored. One effort to promote emotional, as well as material and social, needs for health is the United Nations *Universal Declaration of Human Rights* [3]. The *Declaration* codified a much-enlarged list of biocultural needs of all humans. Article 25 of the *Declaration* makes two points that are especially salient to the topic of love, hope, and human development:

1. Everyone has the right to a standard of living adequate for the health and well-being of himself and of his family, including food, clothing, housing and medical care and necessary social services, and *the right to security* in the event of unemployment, sickness, disability, widowhood, old age, or other lack of *livelihood* in circumstances beyond his control.
2. *Motherhood and childhood are entitled to special care and assistance.* All children, whether born in or out of wedlock, shall enjoy the same social protection.

I have italicized the words of most salience. These words relate to the right to security, the right to a livelihood, and special care for mothers and children. Security, livelihood, and special care are part and parcel of love and hope. To be sure, many other emotional factors, such as empathy, resilience, and self-efficacy, are equally important for healthy human growth and development. It may be argued that hope and love have an underlying primacy and are required to promote these other emotional traits. Perhaps this is why hope and love are so often central themes of literature and art in many human societies.

**Fig. 1:**
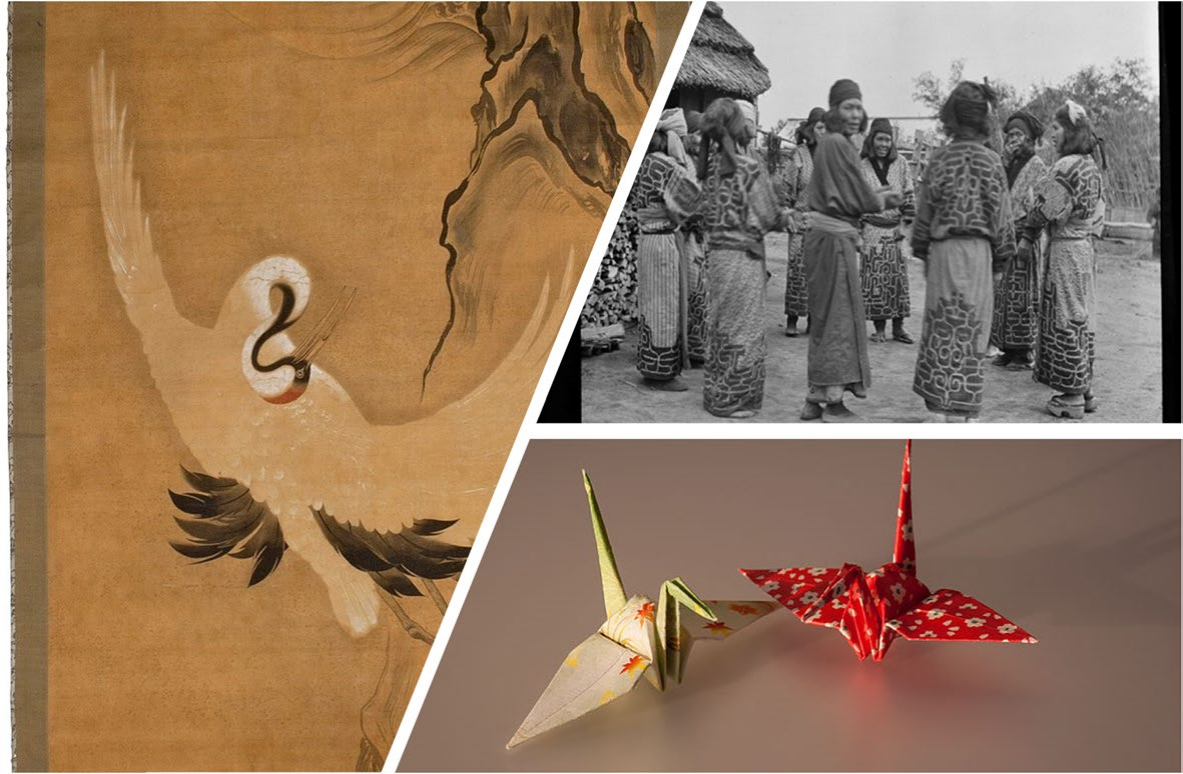
On the left, *Crane*, eighteenth century, by Mitsusuke (1675–1710), National Museum in Kraków. The author died in 1710, so this work is in the public domain in its country of origin and other countries and areas where the copyright term is the author’s life plus 100 years or fewer. Top right, *Crane dance of the Ainu women*, northern Hokkaidō, Japan. Photographer Arnold Genthe, 1869–1942, created/published 1908, Digital Id, agc a05214 https://hdl.loc.gov/loc.pnp/agc.7a05214. No known restrictions on publication. Bottom right, *Cranes* folded in origami paper. Artist Laitche, created: 18 December 2007. This work has been released into the public domain by its author, Laitche. This applies worldwide. In some countries, this may not be legally possible; if so, Laitche grants anyone the right to use this work for any purpose, without any conditions, unless such conditions are required by law

Stories that associate hope with the crane bird (*Grus japonensis* and related species) are one example. Crane mythology can be found throughout history in cultures around the world, from India to the Aegean, South Arabia, China, Korea, Japan, Australia, and North America. In Japan, the crane is one of the mystical or holy creatures and symbolizes good fortune, longevity, peace, and hope. The crane features in traditional Japanese art, dance, and origami (paper folding, Fig. 1). An ancient Japanese legend promises that anyone who folds a thousand origami cranes will be granted a wish by a crane. After World War II, the crane came to symbolize hope for peace and the innocent victims of war through the story of schoolgirl Sadako Sasaki (佐々木 禎子 7 January 1943—25 October 1955) and her thousand origami cranes. Sadako was a victim of the atomic bombings of Hiroshima and Nagasaki by the USA. She was 2 years of age when the bombs were dropped and was severely irradiated. Suffering from leukemia because of the atomic radiation and knowing she was dying, Sadako undertook to make a thousand origami cranes before her death. Following her diagnosis, she survived for another 10 years, becoming one of the most widely known *hibakusha* (被爆者 or 被曝者)—a Japanese term meaning “bomb-affected person.” She is remembered through the story of the more than one thousand origami cranes she folded before her death. Sadako’s brother reports that she folded at least 1300 cranes. After her death, she became internationally recognized as a symbol of the innocent victims of war and remains a heroine to many Japanese girls [4].

The cultural symbolism of hope played an important social role in late nineteenth century Britain. In European Christian theology, *hope* is traditionally considered to be a virtue associated with the grace of God. *Hope* was “naturally" bestowed upon people rather than being earned by work or self-improvement. European artists followed the Greco-Roman tradition of representing *hope* in the personification of young woman.

The impact of industrialization, urbanization, military conflict, civil wars, and revolutionary movements across Europe, combined with laissez-faire capitalism and environmental degradation, during the 1800s caused social upheaval and economic instability. These factors and others led to the long depression or great depression of 1873–1896. This depression had worldwide effects with the greatest harmful impacts in North America and Western Europe, as measured by downturns in the rate of growth of industrial productivity [5]. Productivity in the USA declined by 24% and by 43% in Britain with both the industrial and agricultural sectors suffering. There was unemployment and underemployment, and increased poverty across the British Isles, which then included Ireland. The Bank of England kept interest rates as high as 9% in the 1870s. In 1878, the City of Glasgow Bank in Scotland collapsed, and in 1879, there was famine for thousands of Irish tenant farmers due the high rents and fall in agricultural prices. In 1870, 97% of all Irish farmers were tenants and many were evicted because they could not pay rents. This crisis and the suffering it caused launched the Irish Land War in 1879, which resulted in the reforming Irish Land Acts [6, 7]. Even these acts could not prevent escalating insecurity, violence, hatred, and fear that led, ultimately, to revolution and Irish independence from British rule in 1922.

**Fig. 2:**
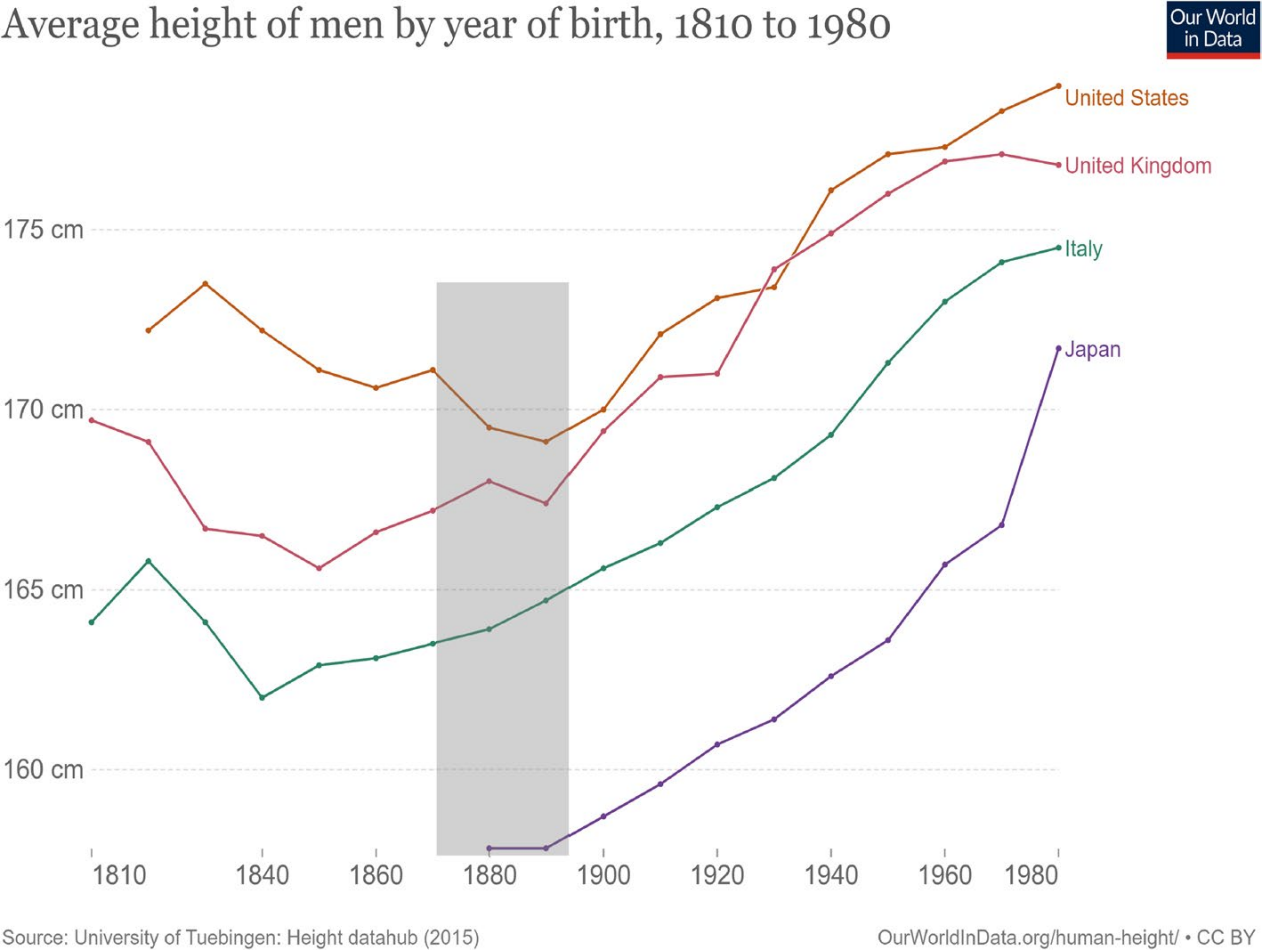
Average height in centimeters of men by year of birth, 1810 to 1980. The data were derived from historical records of the heights of soldiers, conscripts, prisoners, and others. The gray rectangle denotes the time of the long depression of 1873–1896 in the USA and Western Europe. Source of image: https://ourworldindata.org/grapher/average-height-of-men-for-selected-countries?country=GBR~ITA~USA~JPN. Data source: Human Height, University of Tübingen, 2015, Link: https://uni-tuebingen.de/fakultaeten/wirtschafts-und-sozialwissenschaftliche-fakultaet/faecher/fachbereich-wirtschaftswissenschaft/wirtschaftswissenschaft/lehrstuehle/volkswirtschaftslehre/wirtschaftsgeschichte/forschung/data-hub-height.html. Retrieved: 26 March 2023

The long depression induced hardships physically (famine, illness, violence, etc.) and harmed the Social-Economic-Political-Emotional (SEPE) well-being of people globally. Other research has established that SEPE factors are associated strongly with amounts and rates of human growth and maturation [8, 9]. SEPE effects during the long depression were no exception. This may be seen in the changes of height of adult men born before, during, and after the long depression (Fig. 2). In the USA, adult height declines for men born throughout the period of ~ 1870–1885. In Britain (United Kingdom), heights on average stagnate during the same period (slight increase followed by slight decrease). In both countries, after 1885, there is a relatively steep increase in average male heights with each succeeding year of birth. Nearly a century later, there was a decline in height in the 1970s, likely associated with economic recession, social insecurity, fear, and other harmful SEPE factors. The 1973 oil crisis led to the decline of traditional British industries. Inflation in the UK spiked, rising from 9.2% in September 1973 to 12.9% in March 1974, and unemployment also climbed sharply. The government enacted a policy to ration electricity and even so there were frequent power cuts. Another policy enforced a 3-day working week.

In contrast, average adult heights of Italian and Japanese men increased throughout the birth years of 1870–1885. Italy also suffered during the long depression, but the Italian industrial production and Italian average height were very low compared to the USA and Britain. Due to these factors, perhaps, the long depression had no noticeably negative effect on Italian height growth. The trend in male height for Japan is documented only from the year 1880. The effect of the long depression on the Japanese economy was marginal compared with the other countries, as production dropped by only 8% in the period 1873–1896 [10]. The height of men is stable through 1885 and then begins to increase at a rate that is remarkably similar to that of the UK, USA, and Italy, despite the fact that Japanese heights are much shorter in the 1880s. Further discussion of the changes in height by year of birth of men in Japan, Italy, USA, and Britain is given below in the section titled “Secular trends in growth and hope.”

### Harmful SEPE conditions destroy hope and reduce physical growth

**Fig. 3:**
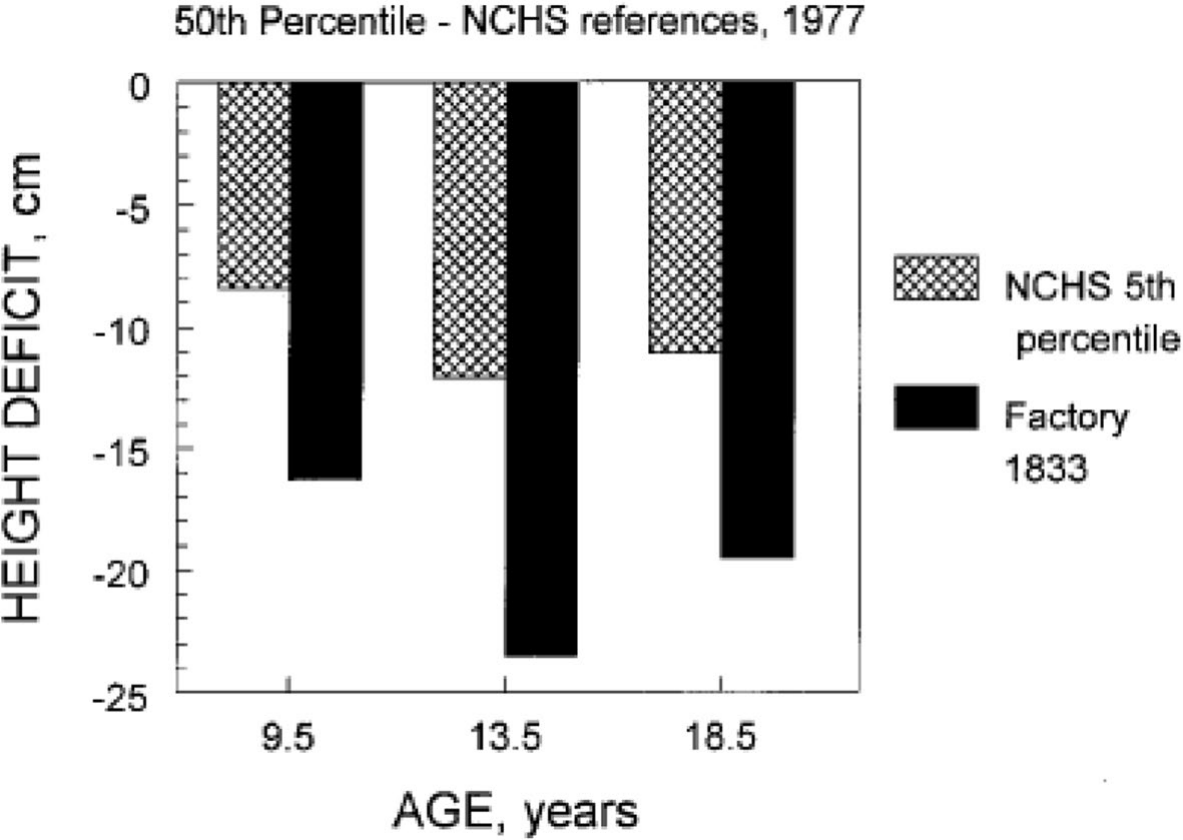
Height of English factory children in 1833 compared with the National Center for Health Statistics (NCHS) references. The heights of the factory children are shown as deficits, in cm, to both the 50th percentile and the 5th percentile of the NCHS references. Original figure by B Bogin, first appeared in Bogin (2021), based on Chadwick’s data as published by Tanner [14]

The social-economic-political turmoil of the long depression provoked emotional responses in the population of Britain. Many people began to question the notion of progress, the existence of God and the virtue of *hope* [11]. New schools of philosophy, especially those based on the writing of Friedrich Nietzsche, saw hope as a negative attribute that encouraged humanity to waste energies on futile efforts. Even before the long depression of 1873–1896, there was rapid and pernicious social and environmental deterioration in British cities and the countryside [12]. There is evidence that the growth of children suffered. As early as the 1830s Edwin Chadwick (b.1800—d. 1890) published data on growth and health of factory children that helped change laws regarding minimum age and working conditions for children. Some of these data are reproduced in Fig. 3, which compares the average height deficit of the English factory children against the international reference data for stature published by the United States National Center for Health Statistics (NCHS) [13]. In this figure, the “0” line represents the 50th percentile height (the “average”) of the reference population. The factory children were 16.3 to 23.5 cm shorter than the 50th percentile of the same aged children in the USA, or England, in the 1970s. Even worse, the factory children were 7.9 to 11.4 cm below the 5th percentile of the NCHS references. In a group of children, an average height below the 5th percentile is an indication of major growth delay and stunting, meaning very low body length-for-age. This magnitude of stunting is usually seen only in children with serious physical/emotional pathology.

Health and nutritional conditions for the factory children were very poor and this undoubtable reduced their growth, but a lack of love by their society and feelings of hopelessness likely further depressed growth. In his 1842 *Report on the sanitary conditions of the labouring population of Great Britain*, Chadwick found that there was a link between poor living standards, the spread of disease, feelings of hopelessness, and poor physical growth. He reported that unsanitary conditions in Britain’s urban slums, into which rural peoples had been forced to migrate due to evictions from their homesteads, had a demoralizing effect on the families affected [15]. The loss of hope in these families was felt by the children, and this further stunted their growth.

### The capacity of artworks to arouse emotions

**Fig. 4:**
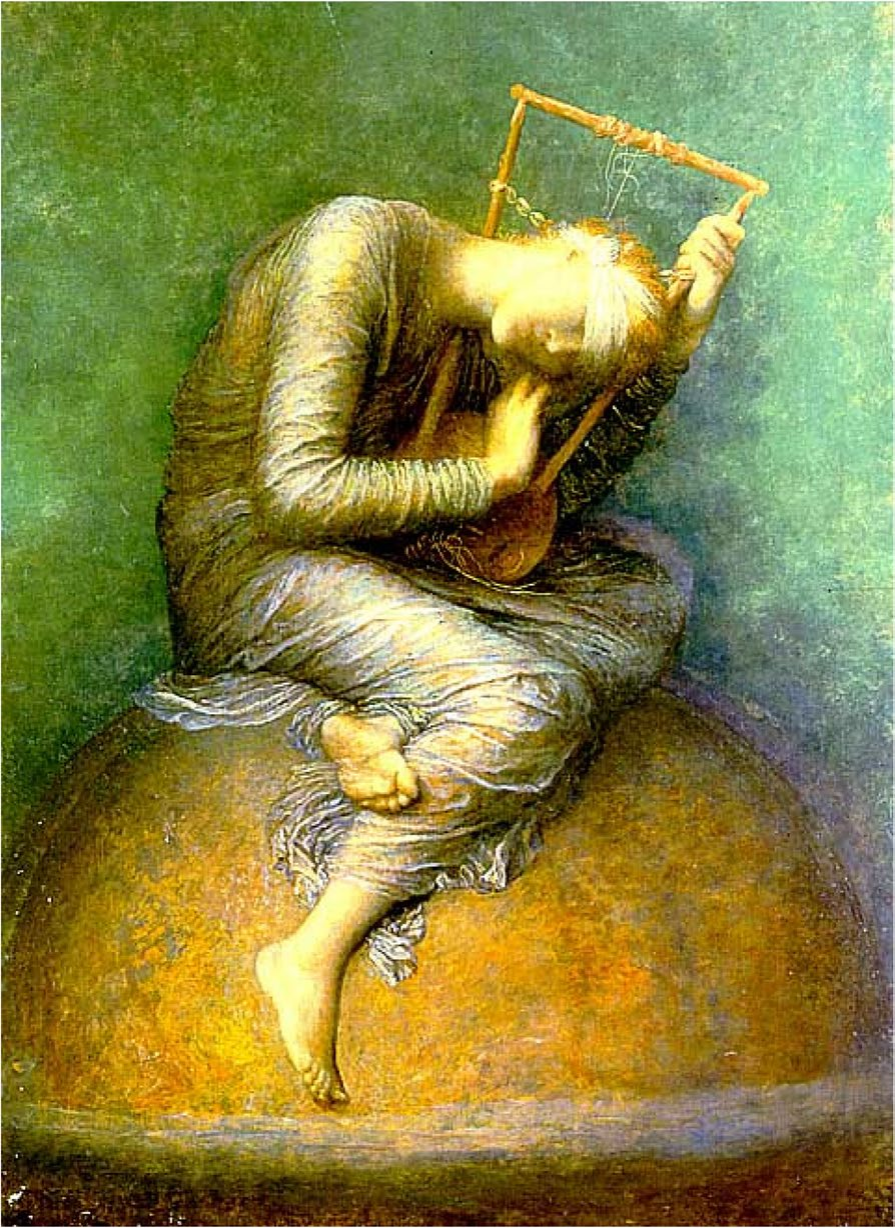
*Hope* by George Frederic Watts (1885) the Watts Gallery, Compton, Surrey. Public Domain. A blindfolded female figure sits on a globe, playing a lyre that has only a single string remaining. The background is indistinct but has one shining star (the star above Hope’s shoulder, enlarge an online image e.g., https://en.wikipedia.org/wiki/George_Frederic_Watts#/media/File:Assistants_and_George_Frederic_Watts_-_Hope_-_Google_Art_Project.jpg)

The artist George Frederic Watts (1817—1904) responded to the demoralizing SEPE conditions of the long depression. Watts produced a series of paintings designed to promote happiness, the most notable was titled *hope* (Fig. 4). Some art historians report that Watts felt that the encroaching mechanization of daily life, and the focus on material prosperity to Britain’s increasingly dominant middle class, were making modern life soulless. A personal tragedy also impacted Watts. In late 1885, his adopted daughter Blanche Clogstoun lost her infant daughter Isabel to illness. Watts wrote to a friend, “I see nothing but uncertainty, contention, conflict, beliefs unsettled and nothing established in place of them” [10, p. 70]. Perhaps to counteract his despair, Watts set out to reimagine the depiction of *hope*. His first *hope* canvas was completed in 1886 and several versions followed over the next decade. The British public reacted favorably, and many cheaply printed copies were sold immediately. Later, higher quality reproductions were available, and these sold well.

The theme of *hope* resonated in British and then American society throughout the next century. Watt’s composition of “hope” influenced several other artists, including Pablo Picasso (e.g., *The Old Guitarist*, painted 1903–04), poets, and a 1922 American film based on the imagined origins of Watts’ *hope* painting (*sic*, “based on a true story…”). In 1954, Percy Collick, a British trade unionist and Labour MP, urged “Labour stalwarts” to attend an exhibition of the *hope* painting because that painting “renewed faith and hope.” Collick’s remarks came after a recent meeting with a Viennese Jewish woman who endured concentration camps and “the terrors of the Nazi War” (quoted in [11]). In 1959, Martin Luther King Jr. delivered his *Shattered Dreams* sermon. In the sermon, King wrote, “There is much truth in George Frederick Watts’ imaginative portrayal of hope in his picture entitled *Hope*. He depicts hope as seated atop our planet, but her head is sadly bowed and her fingers are plucking one unbroken harp string. Who has not had to face the agony of blasted hopes and shattered dreams?” [16].

In the late 1980s, another American minister, Jeremiah Wright, attended a lecture by Frederick G. Sampson in Richmond, Virginia, on the George Frederic Watts’ painting *Hope.* The lecture inspired Wright to give a sermon in 1990 based on the painting. Wright’s sermon included,

“Have the audacity to hope for that child of yours. Have the audacity to hope for that home of yours. Have the audacity to hope for that church of yours. Whatever it is you've been praying for, keep on praying, and you may find, like my grandmother sings, 'There's a bright side somewhere; there is a bright side somewhere. Don't you rest until you find it, for there is a bright side somewhere” [17].

The “bright side” is the one shining star on the background of the *Hope* painting.

**Fig. 5:**
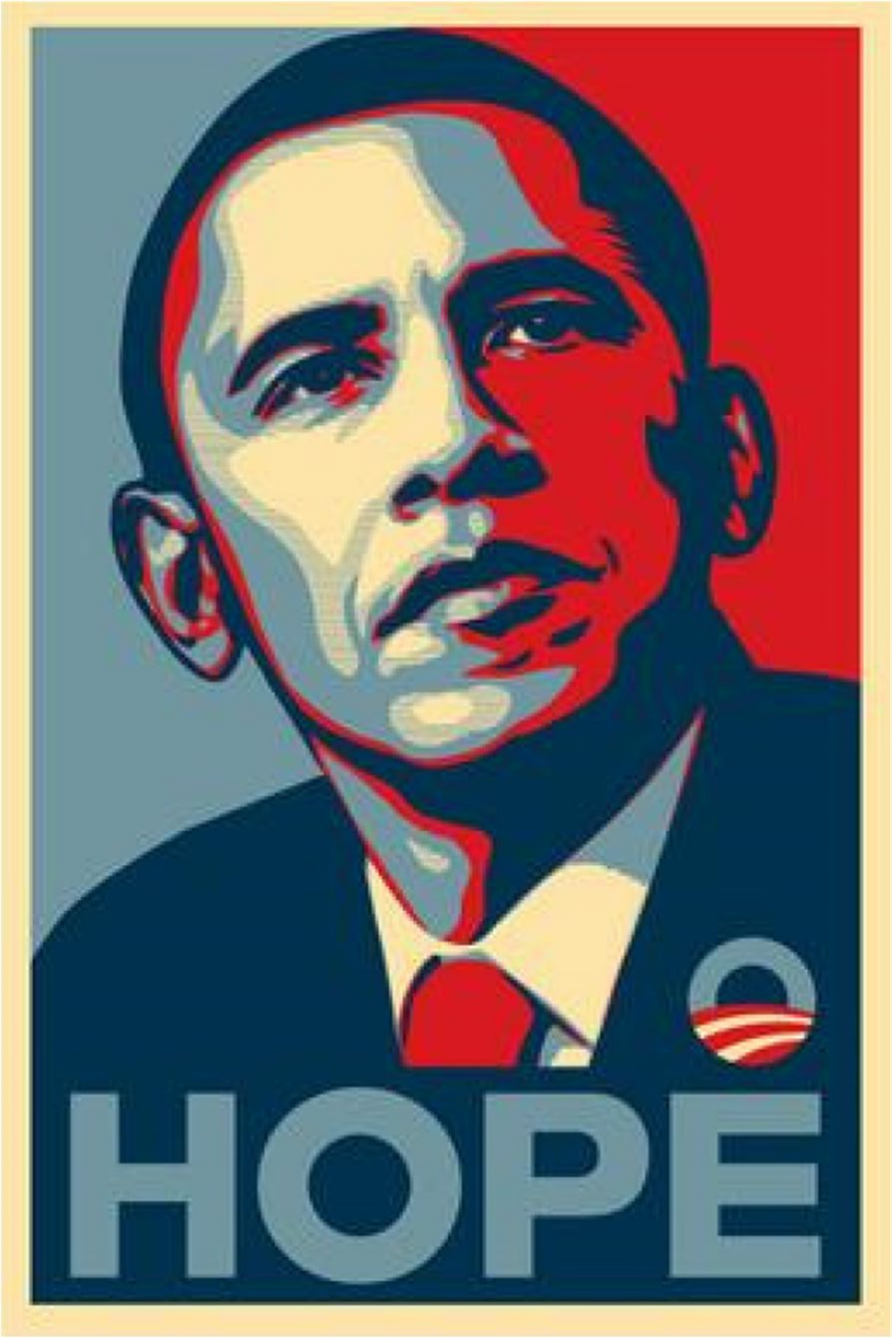
Barak Obama Presidential campaign poster, 2008. Obama Poster Art by Shepard Fairey, based on a photo taken by Mannie Garcia for Associated Press. Image from https://en.wikipedia.org/wiki/File:Barack_Obama_Hope_poster.jpg. Reprinted under the Fair Use statures of United States copyright law

A law student named Barack Obama was in the audience the day of Wright’s sermon. As the presidential candidate in 2004, Obama delivered his campaign address and changed Wright’s “Audacity to hope” to “Audacity of hope.” This became the title of Obama’s second book of memoirs [18]. The “Audacity of Hope” also represented Obama’s 2008 presidential campaign via his “Hope poster” (Fig. 5).

## Hope and love are the fundamental basis of human biocultural reproduction

All human beings, especially, pregnant women, mothers, and young people, need the type of support and care that comes from human systems of healthy biocultural reproduction. The concept of *biocutural reproduction* is defined as, “…the set of marriage and kinship-based rules for extra-maternal cooperation in the production, feeding and care of offspring” [19]. This definition of extended family care of women and their offspring applied to all people during the 99% of human history when our ancestors lived in anthropologically traditional societies, that is, societies based on economies of foraging, horticulture, pastoralism, and pre-industrial farming. Post-industrial and urbanized societies also need healthy biocultural reproduction and assure this via newer forms of assistance from extended family, friendships, paid or unpaid carers, and governmental institutions (schools, health care systems, social care systems, etc.). In both traditional and modern post-industrial societies, “it takes a village to raise a child” (a traditional African proverb). People need the physical and emotional security of knowing that they will have a diet that meets all nutrient requirements, the security of good water, sanitation and protection from infection, and the security of other material safeguards such as adequate housing and education. These are the minimal requirements for survival. But these alone do not make for a healthy, well-grown person. Infants lacking social interaction and love do not grow well physically and may die, even when given all their physical necessities (see below for evidence).

Human biocultural reproduction is successful only with true well-being, and this happens when young people have hope. Hope comes from opportunities to participate in healthy social and community environments, when the young are part of systems of meaningful informal and formal education, and when the young and their families have realistic expectations for a stable, productive, and meaningful livelihoods. The importance of livelihood was mentioned previously in this review as Point 1 of the *Universal Declaration of Human Rights*. Livelihood is essential for hope. This was emphasized by Klaus Toepfer when he was Executive Director of the United Nations Environment Programme. Toepfer stated in 2001 that people have the, “…basic rights to life, health, adequate food and housing, and traditional livelihood and culture” [20]. The UN does not define “livelihood.” A narrow definition of “livelihood” is the work people do to secure an income and physical necessities. In the social sciences, the concept of livelihood extends to include social, cultural, and emotional needs. One biocultural definition of “livelihood” is, “…the command an individual, family, or other social group has over an income and/or bundles of resources that can be used or exchanged to satisfy its needs. This may involve information, cultural knowledge, social networks and legal rights as well as tools, land and other physical resources” [21]. Anthropologists understand that a livelihood is not only a way to make a living—it is a way to make living meaningful. Indian anthropologist Rashmi Rekha Tripathy reviewed research on the social science concept of “livelihood” and wrote, “…there is a moral or cultural dimension to livelihood as well as a material dimension: livelihood involves not simply the satisfaction of material needs it also involves the satisfaction of emotional, spiritual and intellectual needs” [20, p. 1]. Tripathy conducted fieldwork among Juang tribal agriculturalists in Odisha State, India. Tripathy found that changes to the means of financial livelihood due to migration from the village to urban areas not only impacted the people’s economic status but also their socio-cultural life, including their family and kinship networks, religious rituals and festivals, and the marriage system. These are part of the definition of biocultural reproduction given above. Healthy environments for biocultural reproduction and livelihoods ensure emotional security and provide hope to people as much as they provide physical needs.

## Healthy human growth is a “love story”

Readers of this article likely have received and given much love during their lives. At the 2009 symposium, “Origins of Altruism and Cooperation,” Walter Goldschmidt is quoted as stating, “You talk about cooperation and altruism but what you really mean is LOVE. We shouldn’t be afraid to use the word LOVE. That is what makes us truly human” (from the dedication to the book from the symposium [22]. “Love” and “hope” have many meanings, and I focus only on those that relate to biocultural reproduction and human growth. The primatologist Allison Jolly wrote that humans have “,,,few, much loved offspring…” [23]. “Love” in this context refers to the prolonged care that primate mothers lavish on their offspring. A specifically human expression of love and hope is Robert LeVine’s [22, 23] proposal that all human parents have a universal evolutionary hierarchy of goals for their offspring. LeVine’s﻿ parental love and hope manifests as two goals: (1) encourage the survival and the health of offspring and (2) develop offspring into self-supporting adults with culturally specific and acceptable beliefs and behavioral norms. Many anthropologists and others have elaborated on Jolly’s and LeVine’s version of love and hope [24–28]. In particular, Sarah Hrdy builds on Goldschmidt’s work and shows that love and hope manifest in the emotional and physical commitment that many people must make to successfully support a pregnant woman, her infant, and her older children, juveniles, and adolescents. Goldschmidt called the need for this commitment “affect hunger,” which is, “…rooted in biology and emerges with culture” [27 (Goldschmidt, 2006, p. 141]. To satisfy our affect hunger, we humans must have nurturant love from others to complete our physical development and provide the hope needed to bring us to culture. Infants and children deprived of nurturant love and hope often die and those surviving do not grow well in body or brain. They never become healthy, cultural persons.

## A lack of love and hope is deadly

Psychosocial short stature is a well-studied condition that shows how the neuroendocrine system mediates the relationship between psychological-emotional factors and physical growth. Psychosocial short stature is a clinical condition of slow growth or growth failure between birth and adulthood that cannot be ascribed to an organic problem with the person, but rather to behavioral disturbance and emotional stress in the environment in which she/he lives. Another symptom is delayed puberty. A diagnosis of psychosocial short statue is confirmed when the infant, child, or youngster is removed from that environment and growth, development, and maturation are spontaneously restored [29, 30]. Saenger and colleagues [31] showed that changes in growth rate of children with psychosocial short stature are not associated with caloric intake. Rather, many behavioral and emotional factors can lead to psychosocial short stature, and a history of abuse, neglect, and/or emotional deprivation is common. The key proximate cause is hormonal. In a review of his clinical experience with psychosocial short stature Rappaport [32] stated that “…the most consistent biological finding was the decrease of circulating somatomedin activity…”. Somatomedin is now called IGF-1 (insulin-like growth factor-1). IGF-1 is the most potent endocrine hormone for linear growth. Children suffering from affective emotional deficiency and psychosocial short stature may also have reductions and derangements in the physiology of other neuroendocrine markers, such as melatonin, serotonin, β-endorphins, and adrenocorticotrophic hormone (ACTH). Derangement of tryptophan metabolism (an essential amino acid) is also known [33]. Each of these are regulators of physical growth.

As early as the year 1701, there was clear evidence of a relationship between the psychosocial environment and human development. This evidence, published in *Gerhardts Handbuch der Kinderkrankheiten* (Gerhardt’s Handbook of Pediatric Diseases) and reprinted by Peiper in 1955 [34], consisted of the percentage of infants admitted to foundling homes who died while in care. The data are reproduced here in Table 1 in descending order of the percentage of infants dying. In Irkustk, Siberia, Russia, 100% of infants entering the foundling home died, but no date was given by Peiper. In Dublin, Ireland, for nearly all the eighteenth century, 98% of infants died in the foundling homes. The percentage of deaths decreased, generally, over time, but remained 50% or higher except for the cities of Prague and Bordeaux. Those two cities had foundling homes and also placed some infants with caregivers in the rural countryside. The Prague infants seemed to fare worse there and the Bordeaux infants slightly better. Peiper’s book contains several comments on the “clean conditions” and “good diet” provided in the foundling homes, nevertheless so many of the infants died. A lack of adequate psychosocial stimulation, to use the WHO phrase, or more simply put, a lack of love, seems to have been a contributing factor to the deaths.

**Table 1:** Infants dying in foundling homes in European cities or nations with two cases of deaths to infants placed into care homes in the countryside. Data from [34]

City or country	Year	Percent dying
Irkutsk	No date given	100
Dublin	I701–1797	98
Petersburg	1772–1784	85
Brussels	1811	79
Petersburg	1785–1797	76
Vienna	1811	72
Paris	1780	69
Paris	1817	67
Moscow	1822–1831	66
Gent	1823–1833	62
Dijon	1838–1845	61
Mons	1823–1833	57
Brussels	1817	56
Belgium	1823–1833	54
Petersburg	1830–1833	51
France	1838–1875	50
Prague	1865	20
Infants placed in the countryside		35
Bordeaux	1850–1861	18
Infants placed in the countryside		15

In the USA, one of the first observations of foundling home deaths was made by Harry Chapin in 1915. Chapin, a physician, visited 10 orphanages and hospitals in the New York City region that cared for abandoned infants. These institutions provided an acceptable level of care in terms of hygiene and feeding, yet in 9 of the 10 orphanages all the infants under 2 years old died. In 1942, Harry Bakwin proposed that the cause of this extraordinary mortality was emotional deprivation. In the first half of the twentieth century, the medical community in the USA believed that “excessive” physical contact of an infant and its caregivers was deleterious. In the orphanages and hospitals, where the staff were likely to be overburdened with many infants, physical contact was reduced to a minimum. Bakwin believed that the deprivation of physical contact led to a negative emotional state, growth failure, and death.

René Spitz (1887—1974) carefully investigated the causes of poor growth experienced by emotionally disturbed infants and children [35]. His studies focused on orphans confined to foundling homes and other institutions. Spitz quoted a diary entry of a Spanish Bishop who wrote in the year 1760, “*En la Casa de Niños Expositos el niño se va poniendo triste y muchos de ellos mueren de tristeza*” [In the Children’s Home, the child becomes sad and many of them die of sadness]. Spitz compared the development of infants in one foundling home with infants raised in the nursery of a penal institution for delinquent girls. Inmates of the latter facility were the natural mothers of the infants. Both institutions provided an acceptable standard of housing, sanitation, medical care, and diet for the infants. The children in the penal nursery had more physical and social stimulation, due to their full-time care by their own mothers, or full-time substitutes. In the foundling home, care was provided by one nurse, and infants were confined to their cribs, without human contact, for most of the day. Over the 2 years of study Spitz found that the foundling home children became progressively delayed in their physical and mental development compared with the nursery infants and a control group of home-reared infants. At about age 3 years, the foundling home children had average heights and weights expected for children aged 1.5 years. The developmental status of the nursery infants did not differ significantly from a control group of infants raised at home. Moreover, the mortality rate for infants in the foundling home was 37%, while in the nursery group, no child had died. Spitz used the term “hospitalism” to describe the syndrome of poor physical and mental development and high mortality experienced by institutionalized children. It took some time for Spitz’s research to make a significant impact, but eventually a new medical paradigm for infant and childcare was proposed by pediatricians. By the 1950s, Benjamin Spock, the author of an immensely popular “baby care book,” advised parents to hold and cuddle their infants in ways that had been considered indulgent just a few years earlier.

The critical importance of physical contact in early development was the focus of the famous experimental studies conducted by Harry Harlow (1905–1981) and his colleagues [36, 37]. Harlow established several types of rearing environments for rhesus monkeys living in a laboratory. Some infants were raised by their mothers, and other infants were separated from their mothers, but given access to inanimate surrogates. One type of surrogate was a wire frame with a bottle and nipple positioned so that the infant monkey could cling to the wire and feed. Another type of surrogate was a wire frame covered with a soft textured cloth. Infant monkeys preferred to cling to the soft cloth surrogate and would even give up feeding for the opportunity to touch and caress the cloth. Behavioral and emotional development was impaired in the monkeys exposed to both types of surrogates, and the infant monkeys had elevated stress hormone levels, but the impairment was more severe in those with wire-only rather than cloth covered surrogates.

The implications of the research by Spitz and Harlow were applied by Tiffany Field (b. 1942) to the needs of preterm infants. Many preterm infants must be given extraordinary medical care to survive. Poor growth and development of those infants that did survive was a common outcome. Often, the medical care required placement in an incubator that isolated the infant from physical contact with the mother or other caregivers. Field conducted a series of experiments which showed that tactile stimulation could ameliorate much of the poor growth and development. The stimulation could be provided by placing an infant confined to an incubator on a sheepskin fur pad or by allowing the infant to be fondled through “glove hole” access into the incubator. As little as 15 min of gloved touch resulted in 50% faster rates of growth in the isolated infant [38, 39].

## What is more important, food or love?

Green and colleagues [40] reviewed the literature relating to psychosocial short stature, with an aim toward evaluating the role that nutrition and endocrine factors play in the etiology of the disease. It was found that most cases of growth failure in infants (birth to 36 months) were due to malnutrition; these infants were usually denied food by their emotionally disturbed parents or caregivers. Children over 36 months of age were usually not clinically malnourished. Moreover, it was commonly found that both GH (growth hormone) and IGF-1 levels were significantly depressed in these older children. Malnutrition, in contrast, is associated with low levels of serum IGF-1 and abnormally high secretion of GH, so the endocrine profile of the children did not fit with starvation as the cause of their growth failure. Green and colleagues accounted for the growth disturbance in the children with a neuroendocrine hypothesis. They built on the work of Patton and Gardner [41], who proposed that emotional stress may affect some of the higher brain centers, particularly the amygdala and limbic cortex, which are known to control the emotions. Nerve impulses from these brain centers may pass to the hypothalamus where they are transduced into neuroendocrine messages that may affect the production and release of hypothalamic hormones. In this manner, psychological disturbances in the child might be translated into a cutoff of GHRF (growth hormone releasing factor) in the hypothalamus, a halt in GH secretion from the pituitary, and depressed levels of IGF-1 secretion from the body tissues.

Neuroendocrine mechanisms for the relationship between emotional stimulation, growth, and health were confirmed by both experimental studies with nonhuman animals and in clinical human studies. In a laboratory experiment, Meaney and colleagues [42] compared infant rats that were licked by their mothers with infant rats who were not licked. The licked infants had lower levels of so called “stress hormones,” the glucocorticoids such as cortisol, high levels of GH, high growth rates, and even higher scores on tests of learning. In later research, Meaney and colleagues [43] reported direct pathways between physical stress, the release of glucocorticoid hormones, and several central nervous system neurotransmitters which regulate the activity of the hypothalamus and pituitary in humans as well as rats.

In clinical human studies, Skuse and colleagues [44] reported on a group of 29 children with psychosocial short stature and GH insufficiency accompanied with hyperphagia, that is, an excessive intake of food. The researchers found that when these children were removed from their stressful home circumstances, the GH levels spontaneously returned to normal and the hyperphagia ended. Clearly, none of these children was denied food, but they were denied proper emotional care and their growth suffered. Studies with infants and children placed into foster care also find little evidence for a nutritional cause for growth retardation. These foster-care studies do find that one of the first physical changes that occurs with placement is an increased rate of growth in height and weight. Wyatt and colleagues [45] pointed out that most children placed into foster care do not show signs of overt clinical psychosocial short stature or sub-nutrition. Even so, Wyatt and colleagues’ study of 45 apparently healthy and well-nourished infants and children aged 1.5–6 years placed into foster care found more than half experienced clinically and statistically significant catch-up growth in height following placement. This foster-care study shows that even when stature, weight, and food intake appear to be normal, a stressful home environment may be retarding growth. An alternative is also possible, placement into a loving and hopeful home environment stimulates growth.

There are more recent reports of the harmful growth and development consequences of institutional deprivation and emotional neglect/abuse at home, with the dreadful experiences of Romanian orphans being notable [46–48]. These orphans suffered severe psychosocial deprivation prior to adoption, due to the social-economic-political environment imposed under the regime of dictator Nicolae Ceauşescu between 1965 and 1989. Edmund Sonuga-Barke and colleagues have shown that the negative impacts on the orphans included derangements of the hypothalamic–pituitary–adrenal (HPA) axis and stress hormone activity [48]. The association of physical growth with the HPA axis and emotional stress was discussed above.

Even 20 years after adoption into British families providing all needed care and love, the former orphans showed evidence of hypothalamic–pituitary–adrenal axis dysregulation and impaired neurodevelopmental and mental health trajectories. In terms of physical growth, the former orphans did show remarkable catch-up growth in height in the first year after adoption, but had slow growth during the rest of their childhood and adolescence. The former orphans had permanent growth stunting in adulthood due largely to that slow growth combined with an early onset of puberty and early completion of skeletal growth. These consequences were especially severe in girls.

A case study, reported by Magner and colleagues [49], provides a final example of the powerful interplay between emotions and growth. The study is of a 12-year-old boy who suffered growth retardation and delayed sexual maturation following an emotional trauma. The trauma was provoked by an argument between the boy and his stepfather, with whom the boy had a warm relationship. After the argument, the boy verbalized a wish for his stepfather’s death, and the next day the man seriously injured himself falling from a roof. The hospital where the man was recovering sent an erroneous “notice of death” letter to the family’s home which the boy received and read while at home alone. Though the man eventually recovered, the boy began a self-imposed period of food refusal and vomiting. He dropped from 34 to 25 kg in 5 months. At age 15 years, following periods of hospitalization, drug treatment, and counseling his eating behavior returned to normal. But, his growth did not, and at age 17 years, he had the height of a normal child of 11.3 years, a bone age of 13 “years,” and was, essentially, prepubertal in physical appearance. He was given treatment with growth hormone at age 19.3 years, and between ages 20 and 21 years experienced a growth spurt and sexual maturation. Growth in height continued until age 25 years, when the young man reached 171 cm. The authors of this report state that in this patient, an acute, “…psychic trauma induced a deranged hormonal state that persisted for several years” (p. 741). Though malnutrition and drug treatments in the 3 years following the trauma may have also upset the hormonal balance, the boy was behaviorally normal and drug-free for about 5 years before treatment with GH returned his growth and maturation to normal. This case and the others previously discussed exemplify the intimate and powerful influence that emotional factors, such as love, fear, guilt, and hopelessness can have on the human neuroendocrine system and the pattern of human growth.

## Can more love and hope make you taller?

The historical events, human physiological research, and clinical case studies reviewed here show that a lack of love and hope can slow skeletal growth, delay maturation, and even be deadly. The following is evidence that more love and hope can promote greater height and healthier maturation and reduce infant/child mortality.

### Migrants, adoptees, and conquerors—socially upgrading and growing taller

Of particular interest are migrants who escape from poor or dangerous living conditions to find more prosperous general living and socio-economic conditions, usually with improvements in nutrition, health, housing, water, and sanitation [50]. These migrants may be classified as “social upgraders” when successfully integrating into the new host population. Adoptees and refugees from lower-income nations to the high-income nations belong to this group. In contrast, there are “social downgraders,” such as war refugees who are forced into camps or slums. The living conditions of these social downgraders are often terrible and the growth and development of their offspring suffers [51], often due to gross malnutrition and rampant infections. Social downgraders also suffer from social-emotional abuse that leads to mental health difficulties, including post-traumatic stress disorder, depression, self-harm, sleep disturbance, and behavioral difficulties [52]. The daily living conditions in camps and slums impose psychological stressors, such as lack of space and control, violence, feelings of inadequacy, and hopelessness. Social downgraders in the context of migration are not further discussed here, as the harmful consequences of hopelessness on human physical growth were detailed above.

Another group of interest that is considered here consists of migrants who arrive in a new place as invaders, colonial rulers, and other types of political, economic, or psychological oppressors who dominate their host populations. These migrants may be classified as “conquerors.” To compare the effects of being a social upgrader or conqueror, it is useful to test two hypotheses:

1. Cultural and structural assimilation by “social upgrading” migrants into their new host population is accompanied by adjustments in height of their growing offspring toward the median height of the hosts.
2. Migrants using violent, oppressive, or military and colonial conquest is accompanied by height growth of their offspring that significantly surpasses the median height of both the conquered population and the population of origin.

**Fig. 6:**
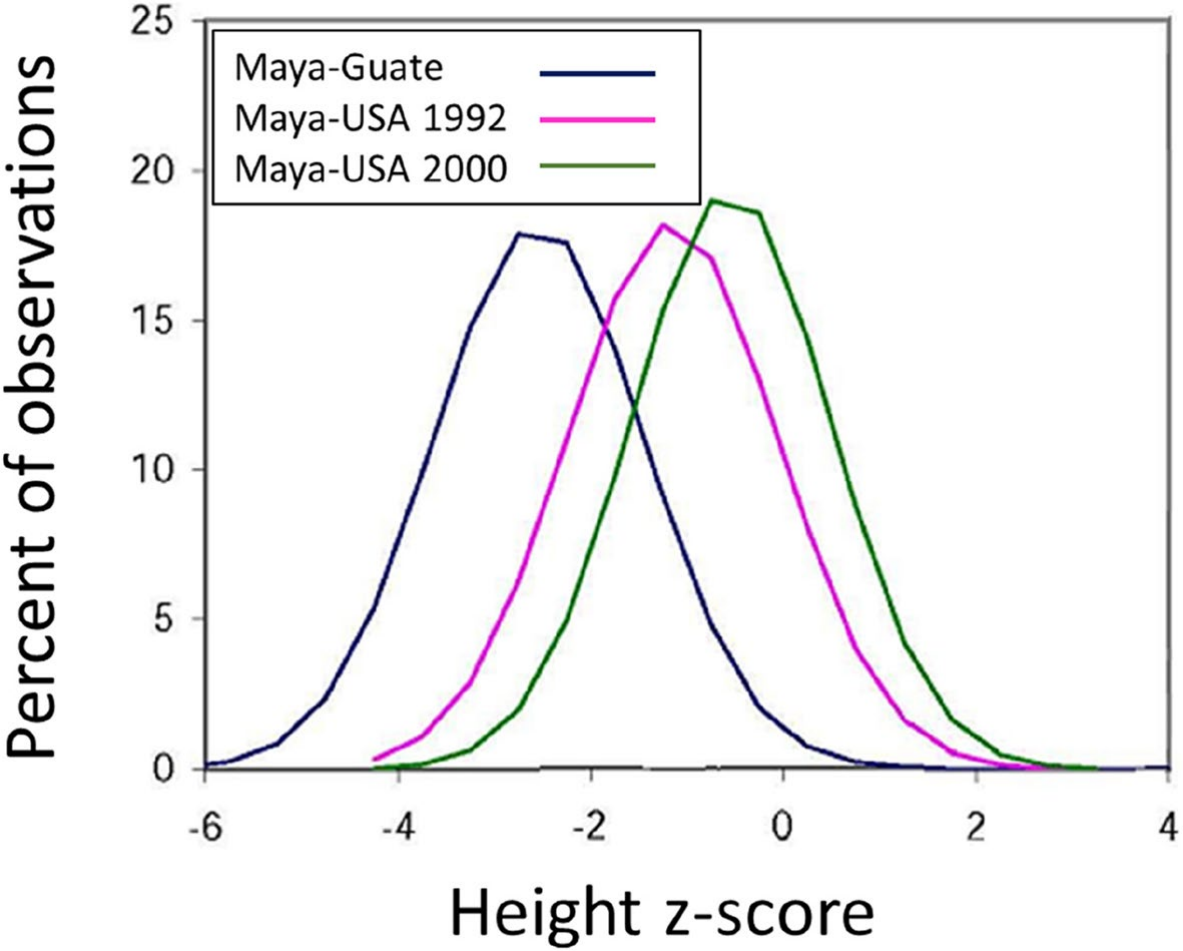
Height distribution in *z* scores of Maya children and juveniles aged 5–17 years old raised in Guatemala (*N* = 1897) in 1998, and in two US locations in 1992 (*N* = 245) and in 2000 (*N* = 444). The width of the three distributions is almost identical despite the much shorter height of the Guatemala-raised sample

Maya refugees from Guatemala to the USA are a test of the first of these hypotheses. A marked improvement in height of children who migrated to the USA may be seen in Fig. 6. The group of children and adolescents aged 5–17 years (*n* = 1897) who were born and raised in Guatemala, measured in 1998, had a mean height − 2.54 *z* scores below WHO standard/reference [53]. The mean height of children and adolescents aged 5–12.99 years (*n* = 245), most born in Guatemala and raised in Florida and in Los Angeles, California, measured in 1992, was –1.15 *z* scores. Finally, the mean height of children and adolescents aged 5–12.99 years (*n* = 444), most born and raised in Florida and Los Angeles, measured in 2000, was only − 0.53 *z* scores below WHO standard/reference. The figure not only shows that migration of the families to the USA affected the mean values of height, but also that migration influenced the entire range of the distribution. The US-born group measured in 2000 increased in height by more than 10 cm within a single generation, but the height difference between the tallest and the shortest within the group remained unchanged as compared with sedentes in Guatemala and those born in Guatemala and migrating in the first months or years after birth. Better nutrition, water, sanitation, and hygiene (WASH), and medical care certainly account for some of the height increase of US-born Maya. But, if these were the only explanations, then we would expect that the lower tail of the distribution, those most suffering from malnutrition, etc., would show the greatest increase in height. This is not the case. Height distributions remain virtually scale invariant, and they simply shift to the right, as a whole.

As explained elsewhere [2, 9], a civil war in Guatemala forced most of the Maya families to migrate. Most adult Maya migrants to the USA aspired to maintain cultural identity rooted in their formative experiences in rural Guatemala, such as Maya language. Maya refugee children, on the other hand, were born or raised in the USA and most learned both English and Spanish simultaneously, fewer of the refugee children learned their Maya language. While Maya values were still strongly emphasized at home, children acquired non-Maya cultural values and behaviors on the streets and in the schools. It is likely that Maya parents loved their children in both Guatemala and the USA, but the migrants had more hope for a better life now and in the future. The increase in height of these socially upgrading and more hopeful children of immigrants moved them closer to the height distribution of their hosts, that is, European-Americans and African-Americans. But the first-generation of Maya-Americans were still part of a migrant community with its own SEPE ecology and still tended to cluster in height. That clustering may explain both their height distribution and their shorter-than-the-reference average height.

Additional examples of a height increase for socially upgrading and more hopeful children of immigrants, for example, from Bangladesh to the UK and from Japan to Hawaii and California, are described by Bogin and colleagues [54].

A relatively new type of migrant are international adoptees (IAs) from low-income countries, adopted by families in high-income countries. Similar to the Romanian orphans discussed above, many of the IAs were institutionalized or placed in foster care prior to adoption. They may have been stunted, wasted, and emotionally scarred by their experiences with maltreating families or neglecting orphanages [55]. At the time of placement with their new families, most of these IAs with delays in physical growth, motor development, and cognitive skills show rapid catch-up of the delays. This was the case for the Romanian adoptees. Some part of the catch-up is certainly due to better nutrition and health care. Another part of the catch-up may be associated with the new social networks of improved emotional care and the host society’s target for taller average height and greater cognitive competences. While the Romanian case shows that the catch-up may be limited and without improvement in adulthood, most other studies of IAs indicate statistically and biologically that the early gains in height and cognition persist into adulthood [54].

Many additional examples of both IAs and adoptees within a nation or community show that adoption is one of the most effective interventions to overcome previous growth stunting and developmental delays. Adoption is certainly more effective than any of the nutrition supplementation, WASH, or education interventions tried in the countries/communities of origin of the adoptees. Adoption, just like the migration of Maya from Guatemala to the USA, places the adoptees in new social networks and leads, in general, to positive community effects and strategic growth adjustments on height, cognition, and emotional well-being [56].

### Community effects and strategic growth

The *community effects hypothesis* posits that the attainment of final height, weight, body composition, and body proportions of individual people arises, in part, from the influence of their bio-social-psychological proximity with members within a social network. These factors influence growth when the brain transduces SEPE factors in the community environment into more-or-less neuroendocrine production of various growth regulating substances. Strategic *growth* may be defined as adjustments to body size or rate of growth that are associated with position in the social hierarchy. Strategic growth adjustments are observed when groups of young people grow larger body size or grow to adulthood at a faster rate to achieve social and reproductive dominance [2, 56]. Strategic growth has been observed in cooperatively breeding mammal species where reproduction is virtually limited to the most dominant female and male individuals [57]. It is also observed in human social groups as part of biocultural reproduction [58]. The catch-up growth experienced by social-upgrading migrants and adoptees are examples of the process of strategic growth in new social-emotional communities, as well as the effects of relief from stress and hopelessness.

Military conquests are a form of migration and also a powerful force influencing love and hope in a society. Following conquest both the vanquished and the conquerors show evidence of community effects and strategic growth changes. Conquerors form the dominant social strata, and they usually take care to ensure that their offspring maintain social dominance. They do not integrate, but rather impose their social networks on their subjects. Colonial Europeans of the nineteenth and early twentieth centuries were not only taller than their native subjects, but also taller than their continental relatives in Europe. White US Americans of the early-to-mid nineteenth century surpassed white Europeans by several centimeters [59]. By 1860, American White men averaged 174.1 cm, while adult men in England averaged 165.6 cm and in Sweden and Norway men averaged 168.6 cm [60]. Greater height for colonists was also true for Dutch white settlers of nineteenth-century South Africa [61] and Australia [62].

Tall stature also prevailed in the children of colonialists during the early twentieth century. Measurements of Dutch children raised in a boarding school in Brastagi, Indonesia, were made between 1926 and 1928 [63, 64]. These Dutch children were similar in height with “tall” US American children measured in 1924 and up to 15 cm taller than German children measured in 1922 or children in Amsterdam measured in 1916 [54]. The Dutch colonial children were, of course, very much taller than indigenous Indonesian children. It is unlikely that the greater stature of the Dutch colonists’ children was due to better nutrition and health compared with their counterparts in Europe. The wealthy of Europe could acquire good diets and medical care. The burden of tropical infections from microorganisms and parasites that are considered responsible for poor growth in many tropical indigenous populations likely also affected the offspring of the new colonists, but these infections do not exist in Europe. Instead, it is proposed that the greater height of European colonists in America and in Indonesia are evidence in support of hypothesis 2 (given above). The greater height of the colonial Europeans was in large part due to their self-perception as the dominant social class with a consequent competitive growth and strategic growth adjustment toward greater average height. The colonists and their children controlled the hope for a better life now and in the future.

## Secular trends in growth and hope

Human biologists and physiological anthropologists define “secular trends” as the change in the mean size, shape, or performance of individuals of a population from one generation to the next. Such trends can be positive (e.g., increasing size) or negative (e.g., decreasing size). These changes may be easy to measure, but what do secular trends mean? Many plausible and fanciful proposals exist to explain secular changes. A few of these are as follows: (1) transportation technology such as bicycles and railroads leading to genetic hybrid vigor as people from formally isolated villages met, married, and produced offspring; (2) changing climate and seasonal effects; (3) the availability and price of sugar or other commodities as a cheap form of food energy; (4) environmental toxicants and endocrine disruptors, such as PCBs, which may accelerate puberty; and (5) the development of public utilities to provide heating—allows greater energy investment in growth vs. keeping your body warm–and artificial lighting–may stimulate growth, somehow. It is also proposed that psychosocial changes in the family, in schools, via media, etc., expose ever-younger children to sexual stimulants that accelerate growth and maturation.

Each of these factors may play some small role, but it is now well accepted that modifications of the social-economic-political-emotional (SEPE) environment leading to transformations in the quality of life are the principle causes of secular changes in growth and maturation. The quality of life may be measured by SEPE variables such as education and literacy levels, food availability/market prices, cost of living, real wages, gross domestic product (GDP), social class and gender stratification/discrimination, rules for voting participation leading to democratization of society, and public expenditures on health. No matter which measures are used, the feelings of safety, security, and a hopefulness for the future are always greater in those populations that have more, and a more equal distribution, of these factors [65].

Human height almost always follows the upward trend of physical well-being, emotional security, and hopeful expectation of a better life. These complex interacting variables and their influence on physical growth are discussed in detail, with supporting literature, in the new edition of my book *Patterns of Human Growth* [2], chapter 7]. The causal relationship between better SEPE environments and greater mean stature is so strong that mean stature itself is used to characterize the SEPE environments of historic and prehistoric populations before the invention of statistics such as infant mortality rates (IMRs), gross domestic product (GDP), literacy rates, or cost of living indices. Some human biologists and economists call the relationship between the S and E (social and economic) components of SEPE environment with height the *biological standard of living* [66–68]. This is a valid perspective, but far too narrow to appreciate the important impact of the political and emotional components that complete the SEPE model.

Pioneering researchers such as René Villermé (1782—1863), Chadwick, Franz Boas (1858—1942) and others from the nineteenth and twentieth centuries emphasized the importance of the SEPE environment as the cause of secular changes but could at best only correlate relationships [2]. Today, we understand many, but not all, of the ways that feelings of chronic stress, love, hope, and other SEPE factors are transduced into neuroendocrine substances by the hypothalamus, the pituitary, the adrenals, other endocrine organs, the skeleton, other tissues and cells, and, even, mitochondria [69–79]. We understand that SEPE factors can exert their influence on human biology via the genome, the epigenome, the proteome, and the metabolome [80–83]. We better understand, and can trace some of the physiological pathways, from chronic exposure to the SEPE “pollutants” of inequality and insecurity to shorter stature, overweight/obesity, both early and late maturation, and behavioral pathology in affected children and adolescents [8, 9, 84, 85]. We appreciate how human biology interacts with high levels of persistent violence to create an ecology of fear and biocultural stress that inhibits healthy growth and development for people of all income levels [9].

We now have a physiological and anthropological understanding of secular trends in stature, such as those depicted in Fig. 2. Where there are declines in adult height prior to 1885, there was also deterioration of SEPE living conditions, related to unregulated industrialization, urbanization, and rising inequality. Parental love for children may not have diminished, but societal “love” for children—in terms of social, educational, vocational, and health support—may have diminished. There was also a loss of hope for a better life. Declines in societal love and in hope repressed growth in stature. Up-turns in height reflect the restoration of hope, whether by belief in an artist’s work or by measurable economic and social improvement. Political change was also a factor. Despite all the environmental harm and economic inequality inflicted by eighteenth and nineteenth century laissez-faire capitalism in Europe and North America, there was also a wave of revolution and civil war starting in 1775 in America and then 1789 in France and continuing to 1917 in Russia. The period 1848–1851 in Europe was especially important as the revolutionary zeal reached the countryside and influenced the peasants, the landless farming tenants, and other low SEPE status rural people [86]. Focusing on this revolutionary period in European/North American history, my colleagues Michael Hermanussen, Sergei Erofeev, and Christiane Scheffler pointed to the associations between the new revolutionary environment of SEPE factors in the cities and the countryside with human physiology and evolutionary biology by writing recently that the,

“…changes in in social and political values within only a few decades, the striving for change and economic supremacy, the pull for aggressive behaviour not just of single persons, but of whole populations – we may mention the political riots and revolutions of that time – now interact with the evolutionarily favoured and conserved neuroendocrine competence for adaptive developmental plasticity. Modern westernised people are competitive. The newly emerged values and behaviours, the striving towards social status and prestige has destabilised the previously rigid feudal dominance hierarchies and conveyed formerly stationary social structures with accepted cues predicting dominance and subordination, into combative structure. The new structures are seductive. They promise personal chances to upgrade in social rank, even with the risk of [there] being costly within group aggression. We consider the rapid secular rise in average body height of the European populations observed during the late 19th and the early 20th century as a result of socially induced permanent overstimulation of the hypothalamic growth regulation” [79].

In their article, titled *The socio-endocrine regulation of human growth*, Hermanussen and colleagues provide the details of the probable pathways from revolutionary changes in social values to hormonal regulation of the skeletal growth (see their Fig. 1). Revolution instilled hope for a better future and people literally grew toward that hopeful future for themselves and their children.

### The secular trend in Japan

There was also revolution and social change in Japan in the nineteenth century. The *Tokugawa Shogunate* had, in 1854, opened Japan to Western commerce and influence. The *Tokugawa Shogunate* was ruled by military leaders but was overthrown in 1868 and was followed by a government formed by politicians, called the *Meiji* government. With the new policies of the *Meiji* government the process of Japanese integration with Western style social-economic-political, and perhaps emotional values accelerated. One of the biggest impacts on the economy that the *Meiji* period brought was the end of the feudal system. According to an anonymous author of the Wikipedia page https://en.wikipedia.org/wiki/Economic_history_of_Japan,

“In the *Meiji* period, leaders inaugurated new, Western-based education systems for all young people, sent thousands of students to the United States and Europe, and hired more than 3,000 Westerners to teach modern science, mathematics, technology, and foreign languages in Japan. The government also built railroads, improved roads, and inaugurated a land reform program to prepare the country for further development … With a more educated population, Japan's industrial sector grew significantly. Implementing the Western ideal of capitalism into the development of technology and applying it to their military helped make Japan into both a militaristic and economic powerhouse by the beginning of the 20th century.”

**Fig. 7:**
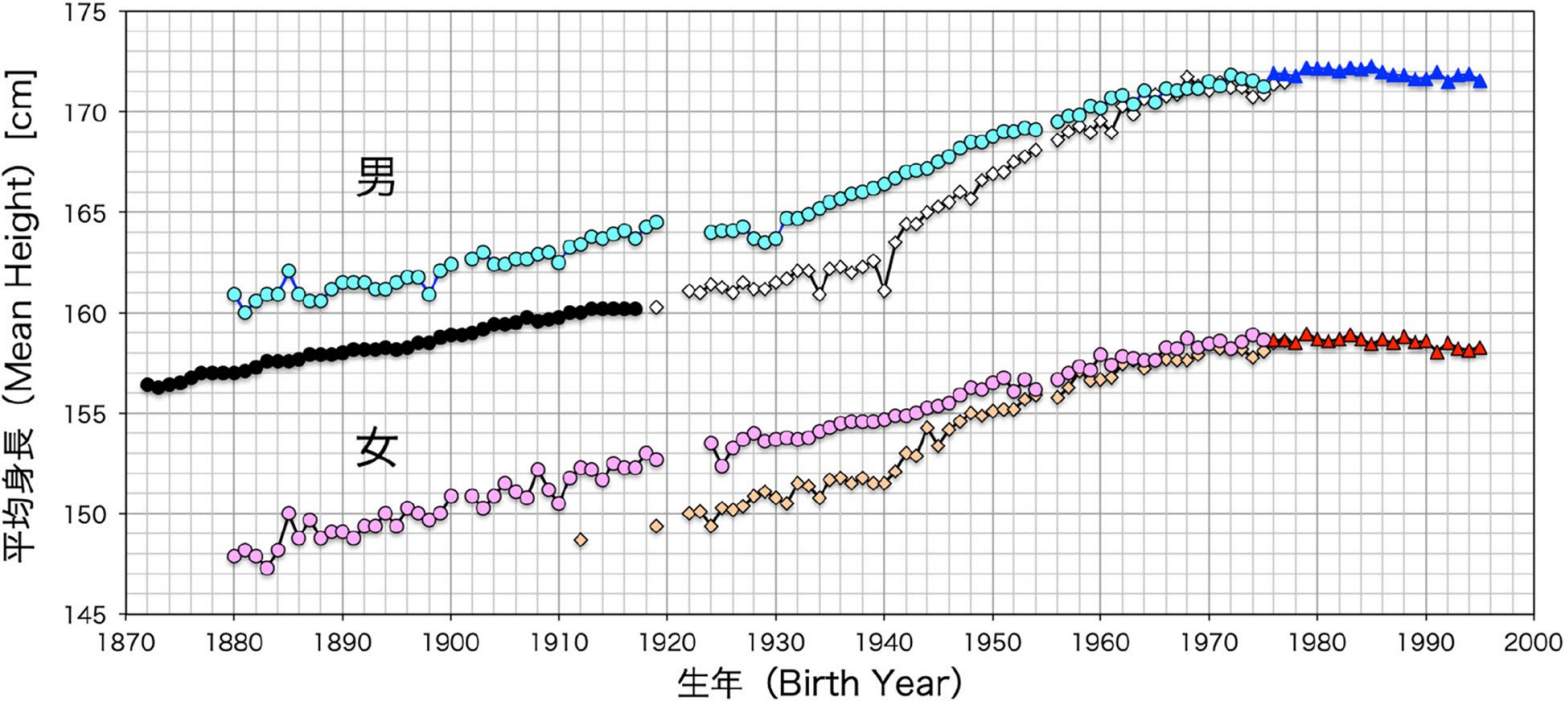
The relationship between the year of birth and average adult height for Japanese born after 1870, based on government statistics. The data for students for the years 1924–1927 and for the general population for the years 1919–1926 are for height measured at ages 21–35 years. All other data are for height measured at age 20–24 years. “The average adult height of Japanese young men and women increased with time, especially in the generations of the 1940s and 1950s after World War II. However, the rate of change has slowed in generations born after the mid-1960s and has stopped in generations born after 1980” (translated from the original Japanese [87]). The upper two plots are for men and lower two plots for women. The symbols indicate:  men  women—high SES 20-year-old students, ● low SES 20-year-old conscripts (men), ◊ men  women—low SES 20-year-old general population,  men  women—post-1975 students and general population. Source: Dr. Makiko Kouchi, National Institute of Advanced Industrial Science and Technology. The figure is reprinted with kind permission of the author

Throughout the *Meiji* period there was rapid industrialization, the development of a capitalist economy, and the transformation of many feudal workers to wage laborers. During the first half of the *Meiji* period, there were labor disputes in the mining and textile industries, mostly in the form of small-scale strikes and spontaneous riots. By the end of the nineteenth century strike action increased. In 1897, a metalworker’s union was established and this marked the beginning of a modern Japanese trade-union movement. With the changes in social structure, education, working conditions, and hope for the future the Japanese people were able to advance through the ranks of society more easily than before. The average height of men (Fig. 7) also advanced at pace with these SEPE changes in Japanese society.

An important analysis by Makiko Kouchi [87] showed that the secular trend in Japan was stronger for people of lower socioeconomic classes than it was for the higher SES groups. The analysis is shown in Fig. 7 with newer data to the year 2000. The data for “students” were based on measurements of university students, who are the upper social class of Japan. “Conscripts” and “general population” are the lower social classes. The data for students for the years 1924–1927 and for the general population for the years 1919–1926 are for height measured at ages 21–35 years. All other data are for height measured at age 20 years. The steepest increase in average height for the general population occurred from the late 1930s to the early 1950s. These are people born 20 years earlier, growing up from the late 1910s to 1920s. After 1975, there were no differences in average height between university students and the general population, in part because a university education was possible for young women and men from the general population. Likely reasons for the strong positive height trend of the general population during the twentieth century were industrialization and urbanization of Japan that brought about major changes in SEPE factors. The changes were especially strong for the lower social classes, including social and political changes due to rural-to-urban migration, universal education, greater democratization, and the breakdown of the power of the monarchy and aristocracy after World War II. These SEPE changes resulted in a reduction of social inequalities, greater socioeconomic opportunities, and greater hope for the lower social classes—the group that experienced the greatest increases in height.

## Conclusion

A famous quote about happiness is, “The three grand essentials of happiness are: Something to do, someone to love, and something to hope for.” These words are often attributed to Alexander Chalmers (1759–1834), a Scottish writer. Some sources attribute the quote to the English essayist, poet, playwright, and politician Joseph Addison (1672— 1719). But the most likely author of the quotation was the American clergyman George Washington Burnap (1802— 1859) and published in Burnap’s book, *The Sphere and Duties of Woman: A Course of Lectures* (1848), in his Lecture IV [88]. The complete quote is on page 99 (page 109 of the online edition of the book),

“The grand essentials to happiness in this life are something to do, something to love, and something to hope for. We all must have something to love. Especially is this the case with woman, whose capacity for affection is much greater than that of man.”

While the book is sexist and couched in the Divine fitting of, “…woman to her sphere” (p.98–99/108–109), Burnap follows the famous quote with statements from,

“…Rousseau, that great delineator of the human heart, which is as true to human nature as it is beautiful in expression; ‘Were I in a desert I would find out wherewith in it to call forth my affections. If I could do no better, I would fasten them on some sweet myrtle, or some melancholy cypress, to connect myself to; I would court them for their shade, and greet them kindly for their protection. I would write my name upon them, and declare that they were the sweetest trees throughout all the desert. If their leaves withered, I would teach myself to mourn, and when they rejoiced I would rejoice along with them.’ Such is the absolute necessity which exists in the human heart of having something to love. Unless the affections have an object, life itself becomes joyless and insipid.”

Another quote from Burnap, page 181/191, “There is no brighter emblem of hope than a vigorous, well developed child. For it we anticipate all the possible enjoyments of this life, it possesses that which is the condition of all satisfaction in anything, a strong physical constitution.” And finally, on page 293/299 Burnap writes, “A bad government paralyzes all enterprise by extinguishing all hope. It puts an end to all invention by taking away all motive. It makes a people idle, vicious, discontented, miserable. Under a good government men work together…”

Unfortunately, the quote ends abruptly as pages 294–295 are missing from the scanned online facsimile of the book.

Even so, we know how good government fosters ways for people to work together for better SEPE conditions, for greater equality in all arenas of life, and for healthier lives.

Burnap knew the essential ingredients of successful biocultural reproduction and healthy human development. To grow well, people—woman, man, child, infant—require love and hope. An important question is: are Burnap’s observations generalizable to different populations or contexts? Based on anthropological literature reviewed in previous sections, such as the work of LeVine, Jolly, Goldschmidt, and Hrdy, the type of love and hope discussed in this article are human universals. The cultural universality of love and hope were identified by Donald E. Brown in 1991 and then again by Brown and others in 2000 [89]. Brown’s list of human universals defines “love” as “affection expressed and felt” and as “attachment.” The universality of hope is listed as the word “hope.”

Another possible limitation of the emphasis on love and hope is oversimplification. Love and hope are but two of the many essential material, biological, social, and emotional needs of people. A lack of any one of these essential needs will impair human well-being. Most of these needs are understood as essential and are codified as such in technical, legal, medical, scientific, and philosophical literature. The essential need for love and hope is less ubiquitous in the literature. The present article argues for greater appreciation and attention to the essential need for love and hope.

Th essential need for love and hope opens the possibility for new research to address several additional questions. Firstly, how can individuals cultivate hope and love in their own lives? An Internet search using the wording of this question returned thousands of web pages with advice and strategies for cultivating love and hope. Readers are welcome to peruse these web pages and glean from them the most appropriate practices for themselves and for others. A second question is: are there any practical applications of this research for healthcare or education professionals? In previous sections of the present article, some applications were described, for example, the critical need to receive touch and face-to-face social interaction by infants in medical isolation and orphanages. With respect to education, a Harvard University webpage notes that, “Very little has been written about how love impacts teaching and learning” (https://www.gse.harvard.edu/news/ed/18/08/what's-love-got-do-it). The website is a review of a book published in 2018 with the title, *Love and Compassion: Exploring Their Role in Education* [90]. The author is Professor John Miller who writes that love is a powerful, motivating force for many teachers and students. Miller’s educational love includes self-love, love of beauty, compassion for others, and a love for learning. The classroom, in Miller’s view, is a community. The community effects hypothesis discussed in the present article has likely implications for human growth and development in both formal and informal contexts of teaching and learning.

A third question: Is it possible to assume that the average height of a group reflects the social, economic, and political factors of that group, while the height of an individual reflects the factor of emotion, in addition to social, economic, and political factors? This question could be the basis of a new program of research. My hypothesis is that social-economic-political-emotional (SEPE) factors, including love and hope, influence the height of both individuals and the group (community) to which they belong. SEPE factors, along with genomics, epigenomics, material, nutritional, and biological conditions, form a complex matrix of interactions (see discussion of the matrix in [2, chapter 8] That matrix of interactions creates, metaphorically, a “biocultural lens” that refracts growth trajectories to create the wonderous plasticity and diversity of human forms.

## Data Availability

The datasets created and/or analyzed during the current study are available from the corresponding author on reasonable request.
